# Behind Closed Doors: The Hidden Realities of Indian Children Living With Alcohol-Dependent Parents

**DOI:** 10.7759/cureus.62989

**Published:** 2024-06-23

**Authors:** Akhil P Joseph, Anithamol Babu

**Affiliations:** 1 School of Social Work, Marian College Kuttikkanam Autonomous, Kuttikkanam, IND; 2 Department of Sociology and Social Work, Christ University, Bengaluru, IND; 3 School of Social Work, Tata Institute of Social Sciences Guwahati Off-Campus, Jalukbari, IND

**Keywords:** social support, stigmatization, sociocultural dynamics, alcohol dependent parent, children

## Abstract

Background: The negative impact of Paternal Alcohol Use Disorder (PAUD) on children's psychosocial well-being is an overlooked issue that warrants more global research attention. Alcohol dependence is often seen as a hidden problem with subtle yet harmful effects, especially on the children of those with alcohol dependence. These children often remain invisible due to their loyalty and unwillingness to disclose their dysfunctional family situation. However, in the Indian context, these invisible children receive limited and insufficient support.

Aim: This study aims to narrate the profound experiences of children growing up with fathers with Alcohol Use Disorder (AUD), highlighting the challenges of invisibility and the support they receive within the Indian cultural context.

Methods: The study used a qualitative narrative research design, with a panel of child psychology experts selecting 12 participants aged 11-13 years. Data were collected through in-depth interviews and a semi-structured interview guide. The data collected were transcribed from the local language into English and analyzed using narrative thematic analysis.

Results: The study revealed how PAUD negatively influences children's experiences and societal norms surrounding family honour and reputation. It also sheds light on the children's reluctance to seek help due to stigmatization, the circumstances that compel them to disclose their problems, and the support they receive to cope with these challenges.

Implications: This study highlights the critical need for culturally specific interventions and support mechanisms in India to address the psychosocial challenges faced by children, emphasizing the importance of societal acknowledgment and strategic interventions to alleviate the detrimental impact of parental AUD on child well-being.

## Introduction

The significant and urgent issue of Paternal Alcohol Use Disorder (PAUD) on children's psychosocial well-being remains underexplored in mental health research. This concern is substantial, as children exposed to PAUD encounter destabilizing factors that lead to adverse mental health outcomes, including psychological trauma, insecurity, and family dysfunction. These conditions can increase their vulnerability to anxiety, depression, behavioral issues, Alcohol Use Disorder (AUD), and, in extreme cases, fatality [1-3]. However, it is crucial to understand children's varied responses to coping with PAUD: some children may suffer profound and long-lasting psychological effects, while others show minimal or transient impacts [4]. This variation underscores the need for detailed research into factors contributing to resilience or vulnerability among these children, such as religious beliefs, socioeconomic status, social support systems, and family structure.

Global studies have consistently shown that a lack of support, compounded by existing vulnerabilities, can predispose these children to similar substance dependencies, thereby perpetuating the cycle of addiction [5-7]. In many cases, mothers are unable to effectively support their children due to managing their spouse's needs, and their psychosocial burdens exacerbate this vulnerability [1]. An unstable and unpredictable home environment impairs a nurturing setting essential for healthy development, often leading children to assume adult responsibilities prematurely, a phenomenon known as "parentification," which can profoundly impact their emotional health, inciting feelings of resentment, anger, and confusion [8]. Early identification of the effects of Paternal Alcohol Use Disorder (PAUD) is crucial for developing culturally sensitive interventions that address the diverse and complex needs of children globally. In regions like Kerala, India, where alcohol is viewed with contrasting attitudes, as sacred in certain rituals, socially accepted yet officially discouraged, the need for robust evidence to guide interventions is particularly urgent, underscoring the widespread nature of PAUD across varied cultural contexts [9]. This study aims to delve deeply into the lived experiences of children in Kerala growing up with fathers suffering from AUD.

## Materials and methods

Research design

This study adopted a qualitative narrative research design to explore children's experiences coping with PAUD. In a narrative research design, individual experiences are placed within a broader social, cultural, and familial context. This method is particularly useful for examining various aspects of a person's life, such as their feelings, ways of coping with challenges, and perception of their environment. This detailed perspective was essential for understanding how these factors influence children's experiences and viewpoints [10]. Given the sensitive nature of the topic and the vulnerability of the participants, building trust and rapport was crucial to collecting data and empowering children to open up by providing them with a secure and supportive environment. Thus, the narrative research design proved more compatible than other approaches.

Recruitment of participants

Participants were carefully selected using purposive sampling from four prominent de-addiction centres in the middle Kerala districts of Idukki and Kottayam, choosing 12 participants to meet data saturation, as recommended by Boddy for achieving saturation in a homogeneous population [11]. The criteria included children aged 10 to 13 living with both parents and having a father with at least two years of documented alcohol dependence who was actively undergoing treatment in a de-addiction center. These criteria were designed to understand the dynamics of paternal alcohol addiction within the family setting and its long-term effects on children's development and familial relationships. Exclusion criteria included any indication of severe neurodevelopmental disorders. The data collection occurred from March to June 2022, providing a focused exploration of the children's diverse yet coherent experiences in the societal context of Kerala.

Tools

Data were collected using a semi-structured interview guide, meticulously developed and validated to ensure reliability. The guide comprised demographic details such as age, educational background, domicile, parents' occupations, and sibling information to comprehend each child's unique background. Also, it included open-ended questions tailored to explore the children's varied experiences and emotional responses to PAUD, probing into feelings of confusion, suffering, isolation, comfort, shared pain, and available support systems. Each question was rigorously tested through a pilot study with three participants, allowing refinements based on initial feedback from them and content validation by two child psychologists from Kerala. This process was supplemented with follow-up probes and debriefing sessions in the actual interviews to guarantee comprehensive data collection and insight into the participants' experiences.

The researchers utilized a go-along interview method, conducting in-depth conversations in settings where participants felt most comfortable, to facilitate data collection [12]. The researchers met the children three times during the data collection: 1. along with facilitators to build rapport, 2. to initiate and continue deep conversations with each child, and 3. to clarify doubts and express gratitude along with the facilitators.

Data analysis

Data analysis was meticulously structured into two phases: thematic and narrative analysis. Initially, thematic analysis was employed using an inductive coding approach to identify and elaborate on major themes emerging naturally from the children's narratives, accurately representing their experiences. Subsequently, narrative analysis was applied to each child's story, employing Labov's narrative structure (1972) to systematically dissect the broader context of their life experiences. This involved analyzing components such as the abstract (summary of the story), orientation (setting the scene), complicating action (main events), evaluation (significance of events), resolution (outcome), and coda (concluding insight), to explore themes like disappointment, love, coping mechanisms, and the impact of PAUD. Each component was explicitly used to align the children’s narratives with established narrative theory, enhancing the depth of understanding and contextual relevance. Also, a reflective journal was maintained throughout the analysis process to document reflections, assumptions, and biases, enhancing the research findings' transparency and reliability.

Ethical considerations

Ethical standards were rigorously maintained throughout the study, adhering to the Institutional Research Ethics Board guidelines and ensuring compliance with ethical principles for research involving human subjects and sensitive information. This study was reviewed and approved by the Research Conduct and Ethics Committee of Christ University, Bengaluru, India (RCEC/00333/06/22). Informed consent was secured from all participants and their guardians, with thorough briefings on the study's purpose, methods, potential risks, and benefits. Anonymity and confidentiality were preserved through pseudonyms and secure storage of interview recordings in a Google Drive account. Participants were informed of their right to withdraw at any time without penalty, and each was provided with a list of mental health resources and support services after interviews to support their well-being.

## Results

Characteristics of the participants

The study included 12 participants, all from a rural area. Participants were grouped into three age clusters: 11, 12, and 13 years, with four participants in each group. This age distribution corresponded to their educational levels in the 5th, 6th, and 7th grades, reflecting age-appropriate educational progression. Their familial backgrounds varied, with siblings and parents engaged in different professions across both skilled and unskilled labor sectors. Despite all participants sharing the experience of growing up with fathers suffering from alcohol dependence, their unique familial and socio-economic contexts enriched the diversity of their narratives (see Table 1).

**Table 1:** Characteristics of the participants The names of the children mentioned in the table are pseudonyms.

Sl. No.	Name	Age	Background
1.	John	13	7th grade. Single child. Rural living. The father is a private employee, and the mother is a homemaker.
2.	Lilly	12	6th grade. One older sibling. Rural living. The father is a government employee, and the mother is a homemaker.
3.	Luka	13	7th grade. Older and younger siblings. Rural living. The father is a plantation worker, and the mother is a laundry worker.
4.	Nathan	11	5th grade. One older sibling. Rural living. The father is a planter, and the mother is a homemaker.
5.	Chris	12	6th grade. Two younger siblings. Rural living. The father is a Mason, and the mother is a saleswoman.
6.	Irine	11	5th grade. One older sibling and two younger siblings. Rural living. The father is a farmer, and, the mother is a homemaker.
7.	Lotta	12	6th grade. Two younger siblings. Rural living. The father is a taxi driver, and the mother is a homemaker.
8.	Milan	11	5th grade. Older and younger siblings. Rural living. The father is a postman, and the mother is a nurse.
9.	Johan	12	6th grade. Older and younger siblings. Rural living. The father is a carpenter, and the mother is a tailor.
10.	Robin	13	7th grade. Single child. Rural living. The father is an electrician, and the mother is a waitress.
11.	Minerva	13	7th grade. One younger sibling. Rural living. The father is a barber, and the mother is a receptionist.
12.	Allen	11	5th grade. Older and younger siblings. Rural living. The father is a chef, and the mother is a hotel housekeeper.

Seeking Clarity Amidst Confusion: The Turbulent Home Image

Children with alcoholic parents endure significant emotional turmoil as they navigate the erratic behaviors and transformations of their fathers, marked by dramatic changes in appearance, language, and emotions. These shifts often result in increased anger and erratic displays of affection, deeply confusing the children. For instance, Lotta compared her father's altered state to a character from a Malayalam movie, saying, "When I detected a foul smell and slurred speech from Dad, I was reminded of Prithviraj's character in 'Pavada' (Malayalam Movie)... I began referring to him as 'Pambu Joy.'" Such observations often leave children seeking explanations for their father's behaviors while witnessing distressing familial conflicts. Lilly shared a painful memory, revealing her fears of her parents separating: "I once asked my mother if she was going to divorce my father and leave me behind." The unpredictability of paternal affection, juxtaposed with aggression, distresses children like John, who noted, "He seemed very loving and caring most of the time, but would come home with a peculiar smell, start arguments, and even physically abuse me without provocation." The emotional burden is further compounded by witnessing the suffering of their mothers. Luka painfully recalled, "One Saturday, I came home to find Mom silently crying while Dad was breaking chairs... She told me she was living for me and couldn't cope with caring for Dad anymore since he had become severely alcohol-dependent." These stories underscore the complex emotional landscape children must navigate in homes disrupted by alcoholism.

Silent Suffering: Bearing the Burden of Secrets

Children coping with a parent's alcohol dependence often face significant social stigma, prompting fears of isolation and other negative repercussions if their family's struggles become known. Minerva relayed her mother's stern advice on secrecy: "My mother urged me to keep my father's alcohol dependence a secret to avoid isolation." This concern about potential social backlash is shared by many, including Chris, who recalled a teacher suggesting to a peer, "Will you become an alcohol-dependent like your father?" This stigma permeates various aspects of their lives, from education, where some teachers unfairly associate children's behavior with their home environments. As Nathan pointed out, "Some teachers link our home environment to our mistakes," affecting their school experiences.

Social interactions can also be painful. Robin overheard a store owner referring to him as "the son of the infamous alcoholic, X." The stigma and fears extend into their homes, where children like Johan find themselves trying to protect their mothers from alcohol-fueled domestic violence: "My dad loves me, but after drinking, he assaults my mom. I try to protect her... After he falls asleep, my mom holds me and weeps." Minerva's experience at a family event, where her mother denied the truth about domestic abuse to maintain appearances, further illustrates the complex layer of betrayal and enforced silence upon these children. She recalls, "At a wedding ceremony, when my aunt noticed a mark on my mom and I explained it was from my dad, my mom lied that she had fallen and insisted I was lying. Afterward, she restricted me from talking to adults." These accounts underscore the profound emotional and social challenges children face in alcohol-dependent households, trapped between loyalty, fear, and the yearning for a normal life.

The Sting of Public Humiliation and Isolation

Children dealing with parental alcohol dependence often face strained social interactions and avoid family gatherings to escape the discomfort of unsolicited advice and critical comments about their fathers' drinking habits. Johan explained, "Although I enjoy family functions, we usually avoid them because people crowd around, advising my mother to admit my father to a reputable de-addiction center and sometimes even accusing her of being incapable of managing the situation." Similarly, Robin recounted an embarrassing incident at a cousin's birthday party where his father's excessive drinking led to a public spectacle: "At the party, my father drank excessively, causing a scene. My uncles intervened, and one mocked my mother, telling her to 'take that man elsewhere' and warning her to keep an eye on me, hinting that I might follow in my father's footsteps." John shared a distressing experience where his father's alcohol-induced paranoia caused him to miss a crucial school event: "My dad, intoxicated, locked my mother and me in our bedroom to protect us from an imaginary threat. I missed a school competition I had prepared for. Later, he apologized, but the damage was done." These experiences reveal the profound impact of alcohol dependence on children's social lives and emotional well-being, leaving them isolated and often embarrassed by their family circumstances.

Shattered Keepsakes and Tear-Stained Faces: The Comfort Found in Confiding

All the children in the study expressed a pressing need to share their painful experiences, particularly during emotionally challenging times. Irine's poignant incident led her to confide in her friend's mother after witnessing her father discard a cherished gift: "When I saw my father discarding the crystal dolls that my best friend had gifted me... I confided everything to her when she asked about my swollen eyes." Milan found support from cousins who helped with homework and meals, citing his mother's frequent illness due to his father's alcohol dependence. In contrast, Allen experienced neglect from relatives, sharing his disappointment when his cousin prioritized watching cricket over helping him with math. Children often found solace in each other, sharing family secrets and providing emotional support. Allen noted the contrasting reactions of his and a friend's mother to their husbands' drinking.

The Power of Friendship: A Secondary Refuge

The emotional support children of alcohol-dependent parents receive from their peers is pivotal, often providing both comfort and practical aid amidst tumultuous home environments. Friends who haven't experienced similar family issues sometimes show greater empathy and support compared to those who have. For instance, after a severe family argument, Lilly confided in her friend Juna about her father's alcoholism and found solace and a temporary home with Juna's family, who embraced her warmly: "Her parents invited me to their home to spend a weekend... They told my parents they consider me a part of their family, as a sister to Juna."

Also, these children frequently found refuge in their neighbors' homes, which offered them a safe haven from the chaos at home. Irine shared how her neighbors provided a protective environment: "I spend most nights at my neighbor's house... That aunt is so loving and compassionate towards us… Sometimes, I wish I were one of their children." However, the situation was complicated by the potential for violent confrontations when mothers sought refuge, highlighting the deep-rooted challenges these families face. As Luka noted, tensions escalated when his mother attempted to find safety with neighbors, leading to confrontational incidents with his father.

Sentiments Towards an Alcohol-Dependent Father: Longing for Normalcy

Children of alcoholic fathers navigate a turbulent mix of emotions, oscillating between hope and despair as they long for the restoration of a normal familial relationship. Lotta exemplifies this daily struggle, praying for her father's sobriety: "Every day, I pray to God for my father to become sober and healthy so that he can return home," she confides, emphasizing her constant hopefulness. During brief moments of sobriety, these children plead with their fathers to maintain abstinence but often face recurring cycles of disillusionment and betrayal when promises are broken. Lilly poignantly captures this painful cycle: "My father always promised not to drink anymore for today, but every night he drank again," a sentiment that evokes the deep emotional toll of repeated disappointments. Meanwhile, children like Robin are painfully aware of the disparity between their familial interactions and those of their peers. He envies his best friend's close relationship with her father, who takes her out for treats and gifts, lamenting, "I wish I had a father like hers, someone who is there and present." This contrast sharpens their longing for a healthier, more engaged paternal relationship.

Unwavering Affection: Love Tinged With Disappointment

Children deeply love their fathers and feel a particularly strong connection when their fathers abstain from alcohol. Chris captures this sentiment: "When my father is not drunk, he is the best father in the world." He treasures moments of sobriety when they can share stories, laughter, and help with homework. This longing for a normal relationship with their fathers also manifests in children's dreams of engaging in everyday activities like going to the movies or shopping, which they see other children enjoying. Irine reflects on the good days, noting, "The days when my father is not drinking are the ones I enjoy the most because he sings old Malayalam songs. We laugh along and sing together; everything seems okay then."

Their optimism extends to a future where their fathers permanently overcome their addictions, leading to more consistent expressions of affection and presence. They envision a life where their families can experience uninterrupted happiness and normalcy. Allen shares a cherished memory that embodies this hope: "On my most recent birthday, my father promised to cook a meal for the entire family. We had a wonderful time, and I looked forward to eating his bread and curry." These moments, where their fathers are fully present and positively engaging with the family, are treasured and feed into the children’s dreams of a happier, more stable family life. They hold onto these experiences as proof that their fathers can change, fostering hopeful anticipation for a future free from the shadow of alcohol.

## Discussion

The study elucidates the complex experiences of children living with alcohol-dependent fathers in Kerala, highlighting how erratic paternal behavior, influenced by alcohol consumption, induces emotional turmoil and confusion. The unpredictable family dynamics, characterized by altered appearances, language, and behaviors of their fathers, perpetuate a sense of chaos, leaving children in a constant search for stability [13-16]. Moreover, the strong social stigma attached to alcohol dependence compels these children to maintain secrecy, exacerbating their isolation and hindering social interactions [17-20]. Despite the familial instability and public stigma, these children cling to moments of paternal affection, which they view as glimpses of hope for their fathers' sobriety [19-22]. In a society like Kerala, where familial bonds are deeply intertwined with societal expectations, these children are pressured to uphold a semblance of normalcy, intensifying their emotional and social challenges [1,21-24].

While this study sheds light on significant aspects of these children's lives, its limitations must be acknowledged [25-27]. The findings, derived from a relatively small sample in rural Kerala, may not be broadly generalizable. Also, relying solely on children’s narratives might present a skewed perspective of the family dynamics impacted by alcoholism. A broader investigative approach, including insights from other family members, educators, and community figures, would provide a more rounded understanding of the impacts of alcohol dependence.

Given these findings, professionals and policymakers need to develop holistic support systems that address both the addiction and its familial impacts [28-30]. This should involve culturally sensitive strategies by mental health professionals to build trust and provide safe spaces for affected children, incorporate successful community-based and peer support interventions, and the training of school personnel to recognize and respond to signs of familial disruptions. Also, these insights should guide the creation of local and national policies, integrating mandatory educator training on substance abuse impacts and increased funding for family-focused support services into public health strategies, enhancing both individual and community resilience. Future research should expand to include diverse geographic and demographic contexts and incorporate multiple perspectives to deepen the understanding of these families' realities. Such an inclusive research approach is crucial for designing interventions that are sensitive to various socio-cultural environments, ensuring robust support for children and families dealing with the challenges of alcohol dependence.

## Conclusions

The current study sheds light on the profound emotional and social challenges faced by children of alcohol-dependent fathers in Kerala. It reveals their struggles with secrecy, public stigma, and the longing for a normal familial relationship amidst erratic and often unpredictable behavior. Despite these challenges, the children's unwavering love and moments of familial affection sustain their hopes for sobriety and stability. However, the research also underscores the importance of holistic, culturally sensitive support systems that prioritize both the immediate and long-term mental health needs of these children. By recognizing the limitations of the current research and the necessity of inclusive, multi-perspective approaches, future interventions, and studies can better address the multifaceted impacts of alcohol dependence on families, ultimately fostering environments that help children thrive despite adversity.
